# Gene expression of NOX family members and their clinical significance in hepatocellular carcinoma

**DOI:** 10.1038/s41598-017-11280-3

**Published:** 2017-09-11

**Authors:** Hyuk Soo Eun, Sang Yeon Cho, Jong Seok Joo, Sun Hyung Kang, Hee Seok Moon, Eaum Seok Lee, Seok Hyun Kim, Byung Seok Lee

**Affiliations:** 1 Division of Gastroenterology and Hepatology, Department of Internal Medicine, Chungnam National University Hospital, 282, Munwha-ro, Jung-gu, Daejeon, Republic of Korea; 2 Department of Internal Medicine, School of Medicine, Chungnam National University, 266, Munwha-ro, Jung-gu, Daejeon, Republic of Korea; 3 School of Medicine, Chungnam National University, 266, Munwha-ro, Jung-gu, Daejeon, Republic of Korea

## Abstract

Nicotinamide adenine dinucleotide phosphate (NADPH) oxidase complex-derived reactive oxygen species (ROS) promote chronic liver inflammation and remodeling that can drive hepatocellular carcinoma development. The role of NOX expression in hepatocellular carcinoma (HCC) has been partially investigated; however, the clinical relevance of collective or individual NOX family member expression for HCC survival remains unclear. Here, we obtained NOX mRNA expression data for 377 HCC samples and 21 normal liver controls from the TCGA data portal and performed Kaplan-Meier survival, gene ontology functional enrichment, and gene set enrichment analyses. Although most NOX genes exhibited little change, some were significantly induced in HCC compared to that in normal controls. In addition, HCC survival analyses indicated better overall survival in patients with high *NOX4* and *DUOX1* expression, whereas patients with high *NOX1/2/5* expression showed poor prognoses. Gene-neighbour and gene set enrichment analyses revealed that *NOX1/2/5* were strongly correlated with genes associated with cancer cell survival and metastasis, whereas increased *NOX4* and *DUOX1* expression was associated with genes that inhibit tumour progression. On the basis of these data, NOX family gene expression analysis could be a predictor of survival and identify putative therapeutic targets in HCC.

## Introduction

Persistent inflammation can initiate and accelerate tumorigenesis^1^. The liver functions to metabolise various endogenous and exogenous substances; however, this constant exposure results in sustained chronic inflammatory stimulation and damage that leads to hepatitis and subsequent remodeling. When left uncontrolled, these processes can cause portal fibrosis, liver cirrhosis, and ultimately liver cancer^2, 3^. Among the primary hepatic malignancies, hepatocellular carcinoma (HCC) is a clear example of inflammation-related cancer, as most cases result from liver damage incurred after prolonged inflammation in the cirrhotic liver^4^. Therefore, it is essential to identify the fundamental factors in the inflammatory cascade regulating the transition from chronic hepatic injury to dysplastic or regenerative nodules or HCC tumours, which may serve as therapeutic targets and prognostic markers in HCC^5^.

Several inflammatory factors may influence HCC prognosis and survival. In this regard, several studies have suggested a role for the NADPH oxidases (NOX) complex, which is mainly involved in the generation of reactive oxygen species (ROS). The mammalian NOX complex family comprises seven paralogues: NOX1-5 and dual oxidase 1/2 (DUOX1/2)^6, 7^. NOX-derived ROS are central factors in oxidative stress and related reduction-oxidase signalling incongruity involved in HCC initiation and development and therefore, considered to be oncogenic factors^8, 9^; however, the functional significance of NOX family members varies by tumour origin^10^. Several NOX studies have suggested close associations between the expression of specific NOX members and HCC prognosis, as well as angiogenesis and metastasis as in other malignancies^10–13^. For instance, high *NOX1* expression has unfavourable effects on recurrence-free survival, whereas increased *NOX4* expression has beneficial effects on both recurrence-free survival and overall survival on HCC^12^. Moreover, another study reported that patients with high *DUOX1* expression had longer recurrence-free and overall survival on HCC^13^. Analysing the effects of the NOX family members on HCC prognosis requires assessing the entire family of NOX genes, using both tumour and adjacent non-tumour tissues and the clinicopathological information of the patients with HCC. However, no study has investigated the effects of the entire family of NOX family genes on HCC prognosis to date.

The present study analysed the expression of all NOX family members in tissue samples from 377 patients with HCC and 21 normal liver tissue samples to determine whether they could be used to predict prognosis. We also analysed the expression of related genes using The Cancer Genome Atlas (TCGA), Gene Set Enrichment Analysis (GSEA), and Database for Annotation, Visualization and Integrated Discovery (DAVID). Significantly, our results validated the relevance of the expression of each NOX family gene to overall survival in the clinical treatment of HCC.

## Results

### NOX family mRNA expression in liver hepatocellular carcinoma (LIHC)

**Figure 1:**
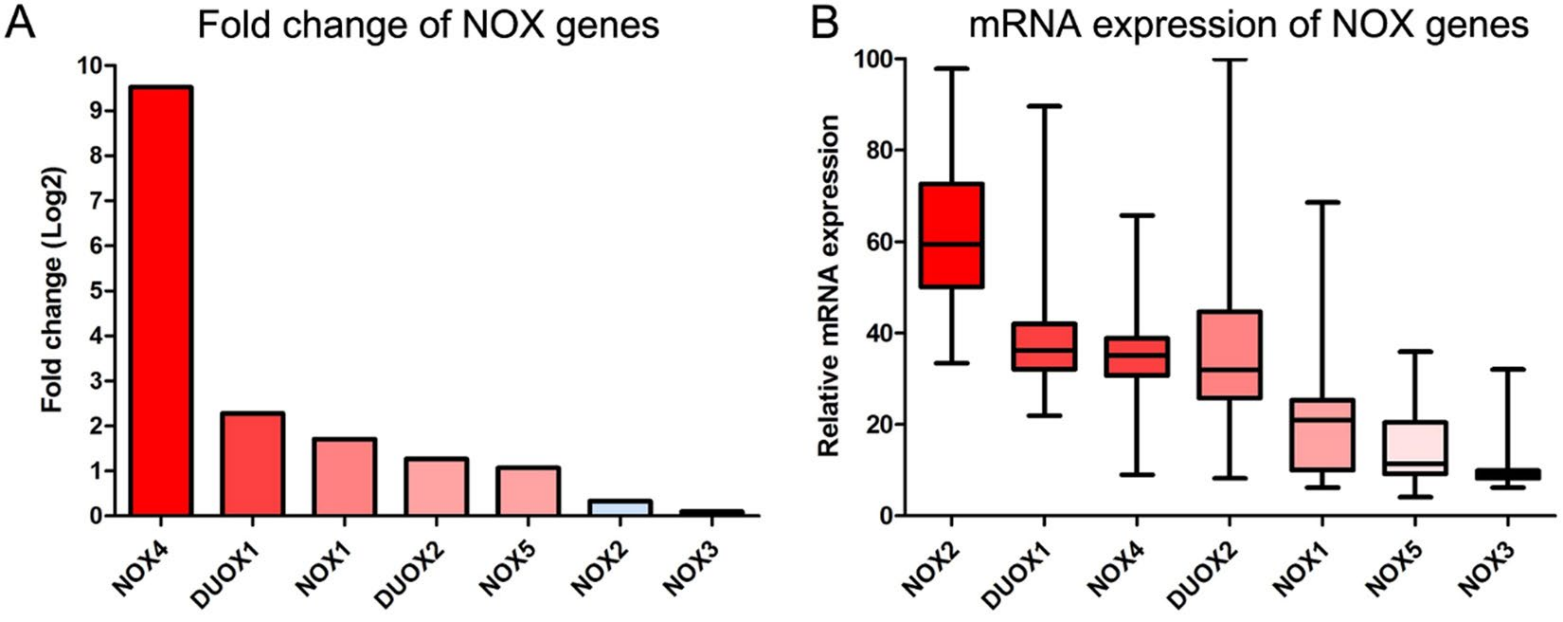
NOX family gene expression in liver hepatocellular carcinoma (LIHC). (**A**) Relative increase of NOX family mRNA expression in LIHC compared to that in normal control tissue. (**B**) Relative expression differences of NOX family genes in LIHC.

Figure 1 shows the mRNA expression levels of NOX family members in LIHC. Interestingly, *NOX1/4/5* and *DUOX1/2* expression was higher in LIHC as compared to that in normal controls, whereas *NOX2* and *NOX3* expression levels were unchanged. Moreover, tumour *NOX4* and *NOX2* expression was markedly higher and lower, respectively, compared to that in the normal controls, the *NOX2* mRNA level was the highest among the NOX family genes. On the other hand, *NOX3* expression was undetectable (Fig. 1A and B). NOX family gene expression showed no significant gene alterations overall (Table 1 and Supplementary Fig. 1). Although no marked differences were observed between sexes, *NOX1* and *NOX4* expression was significantly higher in male and younger patients, respectively (Supplementary Fig. 2). With respect to tumour stage, *DUOX2* expression was significantly higher in advanced TNM stage tumours, however, no significant difference was observed between histological stages (Supplementary Fig. 3).

**Table 1:** NADPH oxidase family of liver hepatocellular carcinoma.

Symbol	Gene name	Aliases	Chromosome location	Fold change (Log)	Alteration (%)
*NOX1*	NADPH Oxidase 1	Mitogenic Oxidase (Pyridine Nucleotide-Dependent Superoxide-Generating)	Xq22.1	1.71	2.7
*NOX2*	NADPH Oxidase 2	CYBB (Cytochrome B-245 Beta Chain), Superoxide-Generating NADPH Oxidase Heavy Chain Subunit, Heme-Binding Membrane Glycoprotein Gp91phox, Neutrophil Cytochrome B 91 kDa Polypeptide	Xp21.1	0.33	4.0
*NOX3*	NADPH Oxidase 3	Mitogenic Oxidase 2, NADPH Oxidase Catalytic Subunit-Like 3	6q25.3	NA	4.0
*NOX4*	NADPH Oxidase 4	Kidney Superoxide-Producing NADPH Oxidase, Kidney Oxidase-1	11q14.3	9.52	4.0
*NOX5*	NADPH Oxidase 5	NADPH Oxidase, EF-Hand Calcium Binding Domain 5	15q23	1.07	4.0
*DUOX1*	Dual Oxidase 1	NADPH Thyroid Oxidase 1, Nicotinamide Adenine Dinucleotide Phosphate Oxidase, Flavoprotein NADPH Oxidase, Large NOX 1, Long NOX 1	15q21.1	2.28	5.0
*DUOX2*	Dual Oxidase 2	NADPH Thyroid Oxidase 2, Nicotinamide Adenine Dinucleotide Phosphate Oxidase	15q21.1	1.27	3.0

### Effect of NOX family gene expression on LIHC patient survival

**Figure 2:**
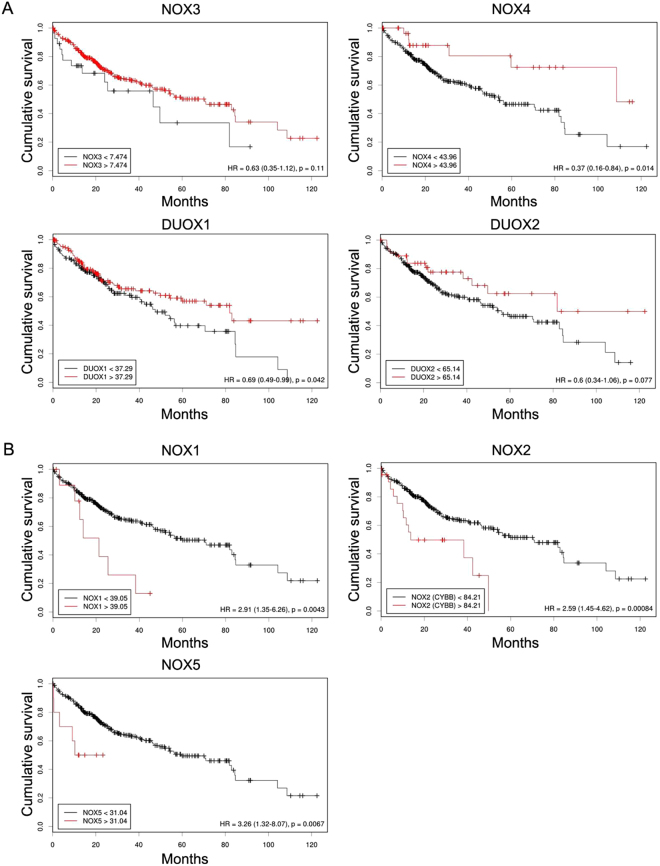
Survival analysis of NOX family genes in LIHC. Kaplan-Meier analysis of the association between mRNA expression of NOX family and overall survival of the patients. (**A**) Cumulative overall survival curve of the patients with high and low expression of NOX3, NOX4 and DUOX1 and 2 (**B**). Cumulative overall survival curve of the patients with high and low expression of NOX1, NOX2, and NOX5. P values were determined by Fisher’s exact test.

Table 2 summarises the clinicopathological data of the patients with LIHC enrolled in this study. To determine the prognostic significance of the NOX family genes in patients with LIHC, we examined the correlations between expression of NOX family members and overall survival of the patients. Initially, Kaplan-Meier curves were used to plot overall survival with mRNA expression, using Cutoff Finder (http://molpath.charite.de/cutoff) (Fig. 2). High expression levels of *NOX4* and *DUOX1* were significantly associated with a better prognosis (Hazard ratio [HR]: NOX4, 0.37 [95% CI, 0.16–0.84]; DUOX1, 0.69 [95% CI, 0.49–0.99]) (Fig. 2A). However, high *NOX1/2/5* expression was significantly associated with a poor prognosis (NOX1, 2.91 [95% CI, 1.35–6.26]; NOX2, 2.59 [95% CI 1.45–4.62]; NOX5, 3.26 [95% CI 1.32–8.07]) (Fig. 2B).

**Table 2:** Clinicopathological information of the liver hepatocellular carcinoma patients.

Feature	Total (%)
**Number**	377 (100.0)
**Sex**	377 (100.0)
Female	122
Male	255
**Age**	377 (100.0)
≤ 60 years	180
> 60 years	196
**NA**	1
**TNM stage**	377 (100.0)
Stage I	175
Stage II	87
Stage III	86
Stage IV	5
NA	24
**Histological grade**	377 (100.0)
Grade 1	55
Grade 2	180
Grade 3	124
Grade 4	13
NA	5
**Vital status**	377 (100.0)
Alive	245
Dead	132
**Child-Pugh classification**	377 (100.0)
A	223
B	21
C	1
NA	132
**Histological type**	377 (100.0)
Hepatocholangiocarcinoma	7
Hepatocellular carcinoma	367
Fibrolamellar carcinoma	3
**Adjacent hepatic tissue inflammation extent type**	377 (100.0)
Mild	101
Severe	19
None	119
NA	138
**Ishak Fibrosis score**	377 (100.0)
0 – no fibrosis	76
1,2 – portal fibrosis	31
3,4 – fibrous septa	30
5 – nodular formation and incomplete cirrhosis	9
6 – established cirrhosis	72
NA	159
**Thrombocytopenia (<150 × 10** ^**9**^ **/L)**	377 (100.0)
Yes	76
No	234
NA	67
**Albumin level, g/dL**	377 (100.0)
> 3.5	217
≤ 3.5	86
NA	74
**AFP (ng/mL)**	377 (100.0)
≤ 20	152
> 20	132
NA	93
**History of hepatocellular carcinoma risk**	
Hepatitis B	105
Hepatitis C	51
Hepatitis B + C	7
Alcohol consumption	118
Non-alcoholic fatty liver disease	18

### Gene neighbours of the NOX family in LIHC

The 100 genes most correlated with NOX family members were identified using GeneNeighbors and classified according to DAVID-based analyses on biological processes, cellular components, and molecular functions (Supplementary Table 1), of which all three showed significant differences (P < 0.05).

#### NOX1

*NOX1* gene neighbours highly expressed in LIHC were primarily associated with metabolic processes (NADP, glucose, and glucose-6-phosphate) when analysed according to biological processes (Supplementary Table 1). For cellular components, *NOX1* was associated with the cytosol and binding-related molecular functions (protein, NADP, and snRNP binding).

#### NOX2

*NOX2* gene neighbours highly expressed in LIHC were associated with the inflammatory response, cell adhesion, and signalling pathways (VEGF, B cell receptor, integrin-mediated, and cell surface receptor signalling pathways) when analysed according to biological processes (Supplementary Table 1). For cellular components, *NOX2* was associated with the plasma membrane, receptor activity (phosphatidylinositol and tyrosine kinase), and binding-related molecular function (MHC class I and actin).

#### NOX3

*NOX3* gene neighbours highly expressed in LIHC were mainly associated with the immune response (defence response and innate response) when analysed according to biological processes (Supplementary Table 1). For cellular components, *NOX3* was associated with keratin filaments and the extracellular region, and receptor activity (G-protein and transmembrane signalling) for molecular functions.

#### NOX4

*NOX4* gene neighbours highly expressed in LIHC were mainly associated with angiogenesis and cell adhesion when analysed according to biological processes (Supplementary Table 1). For cellular components, *NOX4* was associated with focal adhesion and extracellular components, and binding-related molecular functions (integrin and extracellular matrix).

#### NOX5

*NOX4* gene neighbours highly expressed in LIHC were mainly associated with angiogenesis, cell adhesion, and various signalling molecules (VEGF, Rho protein, GTPase and MAPK) when analysed according to biological processes (Supplementary Table 1). For cellular components, *NOX5* was associated with the extracellular matrix and plasma membrane, and binding-related molecular functions (growth factor, integrin, VEGF-activated receptor, and actin).

#### DUOX1

*DUOX1* gene neighbours highly expressed in LIHC were mainly associated with GTPase activity and the mitotic cell cycle when analysed according to biological processes (Supplementary Table 1). For cellular components, *DUOX1* was associated with intracellular cellular components, and for molecular functions, it was associated with binding-related functions (kinesin, GTPase, and NADPH oxidase).

#### DUOX2

*DUOX2* gene neighbours highly expressed in LIHC were mainly associated with differentiation, hydrogen peroxide metabolism, and negative regulation of cell proliferation when analysed according to biological processes (Supplementary Table 1). For cellular components, DUOX2 was associated with the extracellular space and membrane, and binding-related molecular functions (calcium-dependent phospholipid binding, RNA polymerase DNA binding, and CXCR receptor binding).

### GSEA of the NOX family in LIHC

**Figure 3:**
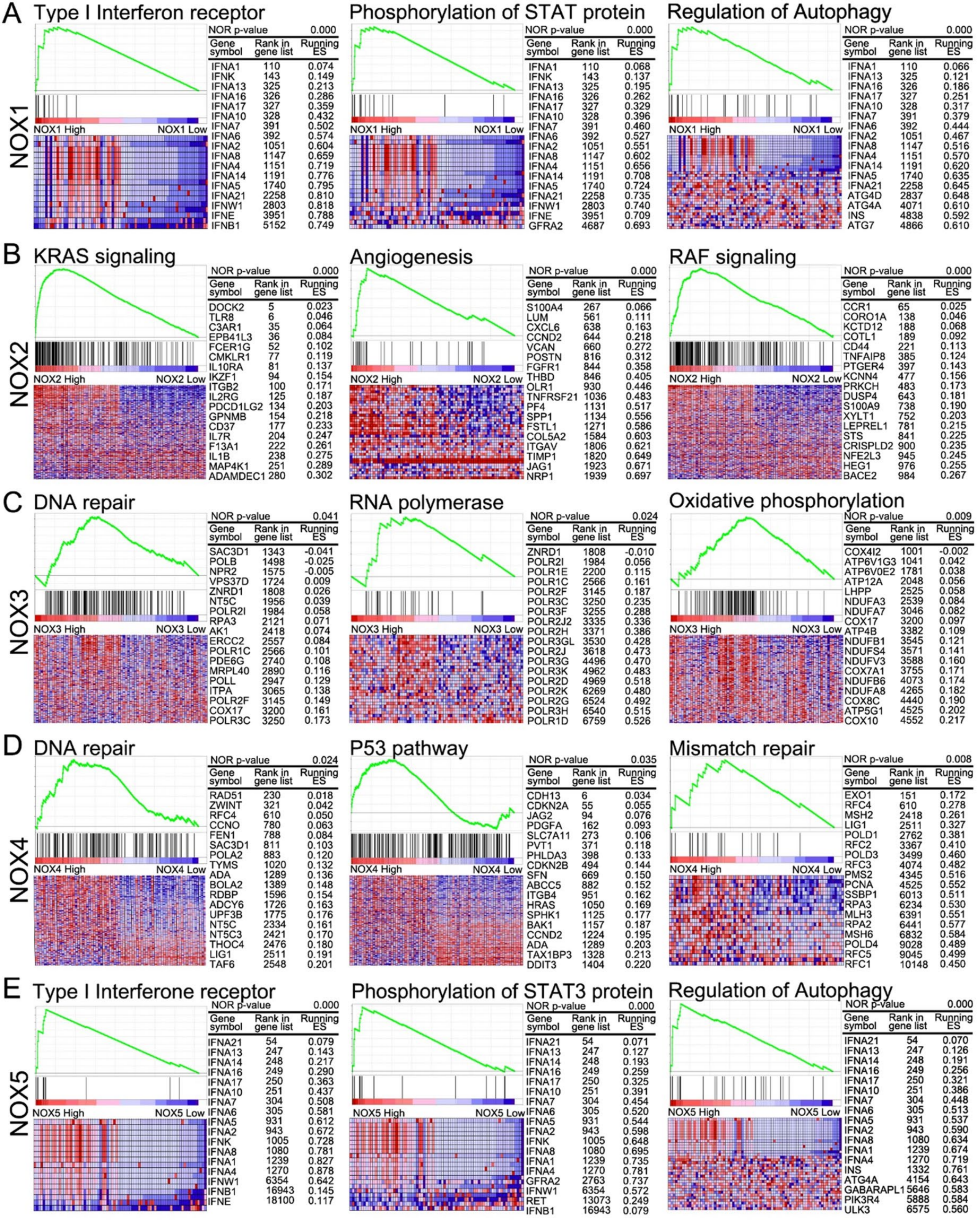
GSEA results for tumours with high *NOX 1-5* expression. Representative GSEA data with p values for (**A**) NOX1, (**B**) NOX2, (**C**) NOX3, (**D**) NOX4, and (**E**) NOX5 are shown.

**Figure 4:**
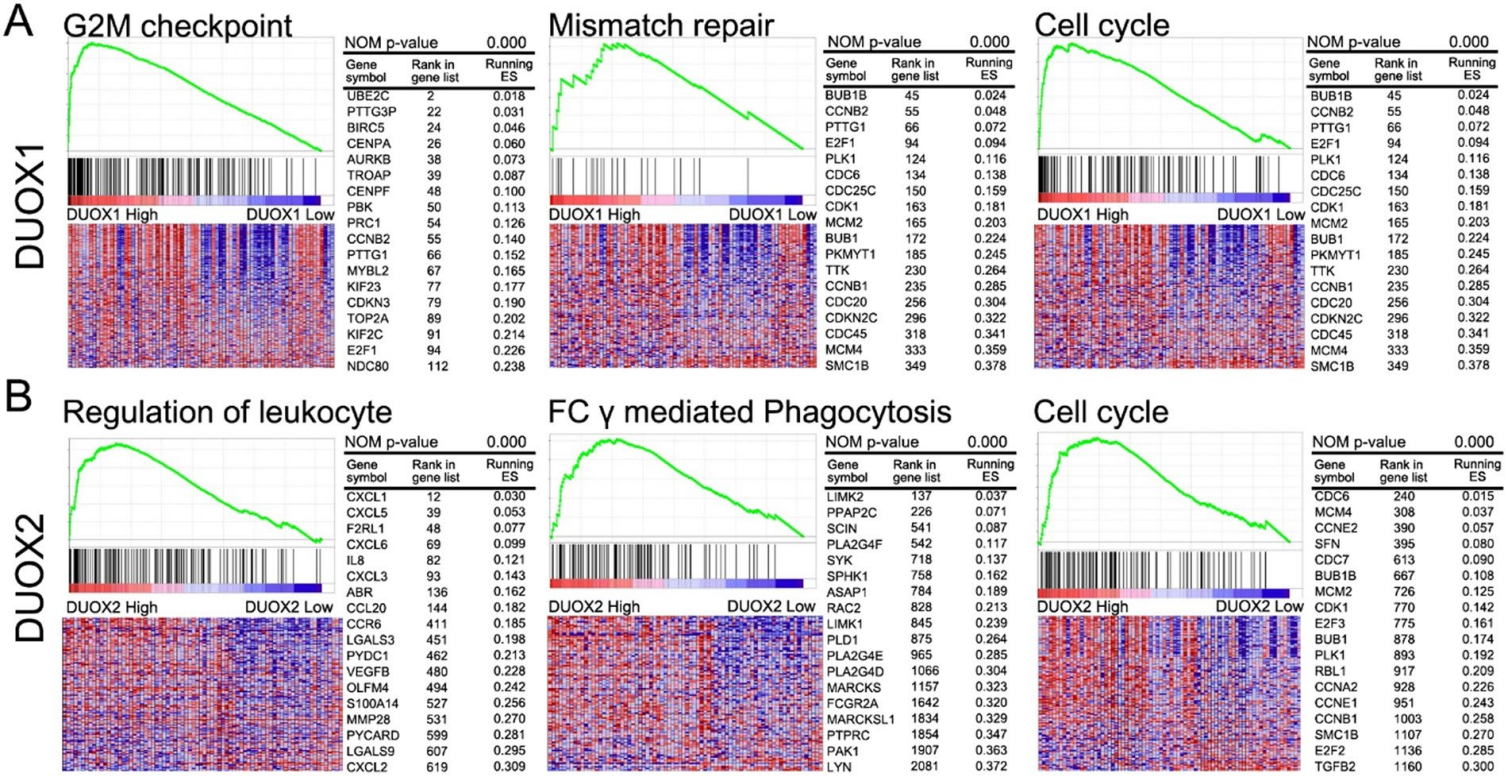
GSEA results for tumours with high *DUOX1/2* expression. Representative GSEA data with p values for (**A**) DUOX1 and (**B**) DUOX2 are shown.

GSEA was performed to identify significantly enriched pathways that differed between the high (top 10%) and low (bottom 10%) NOX-expressing groups based on pathways provided in curated gene set enrichment analysis, KEGG, and oncogenic signatures of gene sets that represent the signatures of cellular pathways often dis-regulated in cancer (Figs. 3 and 4, and Supplementary Table 2).

#### NOX1

In the high-*NOX1* group, the GO terms filament, STAT phosphorylation, mitochondrial translation, and type I interferon, and pathways involving the regulation of autophagy and oxidative phosphorylation, were significantly enriched when compared with those in the low-*NOX1* group (Supplementary Table 2).

#### NOX2

In the high-*NOX2* group, the GO terms immune response, cell activation, and cell adhesion, and pathways involving chemokine, immune response and phagocytosis, were significantly enriched when compared with those in the low-*NOX2* group. Moreover, high *NOX2* expression was associated with oncologic signatures involving EGFR, RAF, and KRAS (Supplementary Table 2).

#### NOX3

In the high-*NOX3* group, the GO terms filament, mitochondrial translation, and ribosome, and pathways involving RNA polymerase, ribosome, and oxidative phosphorylation, were significantly enriched when compared with those in the low-*NOX3* group (Supplementary Table 2).

#### NOX4

In the high-*NOX4* group, the GO terms chromatin and DNA replication, and pathways involving the cell cycle, DNA replication, mismatch repair, p53 signalling, and oxidative phosphorylation, were significantly enriched when compared with those in the low-*NOX4* group (Supplementary Table 2).

#### NOX5

In the high-*NOX5* group, the GO terms type I interferon, filament, STAT phosphorylation, and drug metabolic process, and pathways involving autophagy regulation, fatty acid metabolism, peroxisome, and the RIG-1 signalling pathway, were significantly enriched when compared with those in the low-*NOX5* group. In addition, increased *NOX5* expression was associated with oncologic signatures involving PKCA (Supplementary Table 2).

#### DUOX1

In the high-*DUOX1* group, the GO terms chromatid segregation and cell division, and pathways involving the cell cycle, DNA replication, mismatch repair, and the p53 signalling pathway, were significantly enriched when compared with those in the low-*DUOX1* group (Supplementary Table 2).

#### DUOX2

In the high-*DUOX2* group, the GO terms extracellular matrix, metabolic process, leukocyte migration, and Rho protein signal transduction, and pathways involving extracellular matrix receptor interaction, phagocytosis, and the cell cycle, were significantly enriched when compared with those in the low-*DUOX2* group (Supplementary Table 2).

## Discussion

Several factors associated with hepatic inflammatory signalling are involved in HCC occurrence and progression, including ROS^14^. In mammalian cells, physiologically low levels of ROS play a central role in molecular communication by acting as secondary messengers; however, persistent ROS accumulation can induce inflammatory responses, resulting in genetic instability, chromosomal damage, and tumour development and metastasis^14–16^. Because NOX family members are involved in ROS production and carcinogenesis^17–19^, the present study sought to determine whether these factors were significantly correlated with HCC patient survival.

Previous studies have reported that several NOX subtype proteins are tumour-suppressive or serve as favourable prognostic factors in the liver. For example, high *DUOX1* expression in HCC is associated with prolonged disease-free and overall survival after radical tumour resection; however, this gene is frequently silenced by promoter hyper-methylation in human HCC tumour tissues and cancer cell lines^13^. Furthermore, restoration of *DUOX1* expression significantly inhibits cancer cell proliferation by inducing G2/M phase arrest^20^. As such, it was concluded that *DUOX1* acts as a tumour suppressor in HCC development. In addition, another study has suggested that *NOX1* and *NOX4* are useful prognostic biomarkers after HCC resection^12^. In this study, HCC patients with high *NOX1* or low *NOX4* expression, as assessed by immunohistochemistry, had worse recurrence-free and overall survival rates^12^. Therefore, it was suggested that these patients would benefit from adjuvant treatment after surgical tumour resection, although the underlying molecular mechanisms remain unknown. Similarly, Isable *et al*. reported that *NOX4* may act as a tumour suppressor in the liver, based on data from partial hepatectomy and xenograft mouse models and analyses data from human HCC cell lines and liver tumour tissues^21^. It was concluded that *NOX4* in liver tumour cells acts as a growth inhibitor, consistent with its potential role in counteracting growth factor signals and/or inducing senescence^21^. However, these studies addressed only *NOX1/4* and *DUOX1*. Moreover, only some of these studies discussed the underlying mechanisms involved, especially those pertaining to the cell cycle, and none presented a comprehensive analysis of the underlying mechanisms. Given the lack of information on the relationship between these genes and HCC survival, we investigated the potential of NOX family members as prognostic factors in HCC.

In our study, *NOX1/4/5* and *DUOX1/2* expression was higher in hepatocellular carcinoma tissues than in control normal liver tissues (Fig. 1). Paradoxically, higher mRNA expression levels of *NOX4* and *DUOX1* were significantly associated with prolonged overall survival (Fig. 2A), whereas increased *NOX1/2/5* expression was significantly associated with a poor overall survival (Fig. 2B). These findings are supported by those of several previous studies in which *NOX4* and *DUOX1* expression was associated with a favourable prognosis and *NOX1* expression was correlated with decreased survival^12, 13, 21^.

We performed bioinformatics analyses to verify the effect of NOX family expression on cumulative overall survival in HCC. Notably, *NOX1* expression was significantly associated with metabolic processes, including those of NADP, glucose, and glucose-6-phosphate. Cancer cells often exhibit accelerated aerobic glycolysis to deal with unfavourable situations, primarily hypoxic conditions, and this can distinguish cancer cells from normal cells^22, 23^. In addition, *NOX2* expression was significantly associated with the inflammatory response and VEGF signalling pathway in HCC, consistent with previous reports that VEGF functions as a direct link between chronic inflammation and tumour progression^24^. Especially, the oncologic signatures of *NOX2* expression correlate with EGFR, RAF, and KRAS activity. Moreover, *NOX5* expression was significantly associated with angiogenesis and VEGF signalling, as well as STAT phosphorylation, type I interferon, and autophagy regulation in HCC. The *NOX5* oncologic signature was also significantly enriched for PKCA signalling. Our analyses revealed that expression of both *NOX1* and *NOX5* was associated with increased STAT protein phosphorylation, type I interferon receptor-related gene expression, and the induction of autophagy regulatory genes. Autophagy is a typical survival strategy utilized by tumour cells in unfavourable environments such as hypoxia or hypoperfusion^25, 26^. This process has been reported to induce JAK2/STAT3 phosphorylation and can contribute to cancer cell survival. Lastly, *NOX2* expression was associated with the induction of oncogenic KRAS and RAF signalling, and upregulation of angiogenesis-related genes that contribute to tumour progression, metastasis, and reduced survival rate.

Next, we analysed the genes associated with prolonged survival of HCC patients. A significant increase in *NOX4* expression was found in LIHC tissues compared to that in normal controls, paradoxically, however, *NOX4* expression was correlated with a better prognosis. Indeed, *NOX4* promotes angiogenesis and cell adhesion control^27, 28^; however, genes associated with pathways involving the cell cycle, DNA replication, mismatch repair, and p53 signalling were also significantly enriched in patients with high *NOX4* expression. Previous reports indicate that NOX4 not only promotes angiogenesis via endothelial nitric oxide synthase activation, HIF-1α-mediated angiogenesis, and VEGF expression, but also inhibits the epithelial-to-mesenchymal transition to attenuate liver cancer progression^21, 27–29^. Thus, the functional significance of *NOX4* expression in HCC requires further study.

With respect to the dual oxidase enzymes, *DUOX1* expression was associated with NADPH oxidase activity and the cell cycle, DNA replication, mismatch repair, and p53 signalling pathways, which were responsible for favourable effects on HCC survival, whereas *DUOX2* expression was associated with attenuated cell proliferation, immunological pathways involving Fc-gamma receptor-mediated phagocytosis, and cell cycle regulation, which are associated with a better prognosis. LIHC samples showed higher DUOX1 and DUOX2 expression as compared to non-malignant tissues, and this expression pattern was previously associated with diminished recurrence-free and overall survival^30^. Unlike our present study, *NOX4* expression was not significantly correlated with survival in the previous study^30^. This discrepancy may be due to the relatively large number of patients enrolled in our study (n = 377 vs. n = 107). Moreover, genes associated with mismatch repair, G2M checkpoint, and the cell cycle were also induced in patients with high *DUOX1* expression levels. Similarly, recent genome sequencing and cell cycle analyses demonstrated that *DUOX1* overexpression was associated with cell accumulation in the G2/M phase and cell cycle arrest^20^. In addition, genes associated with leukocyte migration and Fc-gamma receptor-mediated phagocytosis were highly induced in patients with high *DUOX2* expression. In the tumour environment, Fc gamma receptor activity elicits antibody-dependent cellular cytotoxicity or phagocytosis^31–33^. According to these data, *NOX4* and *DUOX1/2* exhibit a tumour suppressive function.

In summary, our data shed light on the clinicopathological mechanisms of NOX family members in HCC, including their diverse roles in inflammatory signalling, angiogenesis, tumour-suppressive and oncogenic gene expression, DNA repair, and the cell cycle control. Thus, NOX genes can positively or negatively affect HCC patient survival through various mechanisms. These findings support the use of a NOX gene expression panel to predict HCC patient survival after tumour resection and suggest that molecular targeting of NOX enzymes may be an effective strategy for HCC therapy.

## Methods

### Gene expression profiles

Level 3 mRNA expression and clinical data from 377 LIHC and 21 normal control samples were obtained from the TCGA data portal, and RSEM_genes_normalised RNA-Seq data were acquired from Firebrowse for analysis of gene expression. All of these datasets downloaded during and analysed during the current study are available in the TCGA data portal (http://tcga-data.nci.nih.gov/tcga) and Firebrowse (http://firebrowse.org/?cohort=LIHC&download_dialog=true).

### Analysis of mRNA microarray data

The data from TCGA portal were analysed using R software (v.3.2.5; http://www.r-project.org). The Rank Normalize module in GenePattern (http://broadinstitute.org/cancer/software/genepattern) was used to normalise the chip data. The data for the fold change of NOX family genes in LIHC compared to normal controls were derived from Firebrowse (http://firebrowse.org/viewGene.html?gene=NOX1), where the gene name in the web address for Firebrowse could be substituted by the name of the gene of interest. Illuminahiseq_maseqv2-RSEM_genes_normalised RNA-Seq data were acquired from Firebrowse (http://firebrowse.org/?cohort=LIHC&download_dialog=true) and analysed using GeneNeighbors, a module programmed in GenePattern, to select genes closely related to NOX family genes^34^. cBioportal (http://www.cbioportal.org/) was used to analyse changes in the expression of NOX family genes in LIHC.

### Functional enrichment analysis

First, 100 differentially expressed genes (DEGs) analysed using GeneNeighbors were imported into Database for Annotation, Visualization and Integrated Discovery (DAVID) (http://david.abcc.ncifcrf.gov/) for gene ontology (GO) functional enrichment analyses. After the data for DEGs were submitted, they were converted and analysed using the gene accession conversion tool and functional annotation tool in DAVID, respectively. Gene set enrichment analysis (GSEA) was performed to find enriched mRNAs predicted to have a correlation with pathway in C2, a curated gene set and the Kyoto Encyclopedia of Genes and Genomes (KEGG), and in C5, a gene set that contain genes annotated by the same GO term, and in C6, oncogenic signatures of gene sets that represent the signatures of cellular pathways that are often dis-regulated in cancer. Especially, GO analysis encompassed three domains: biological processes, cellular components, and molecular functions. P < 0.05 was considered to indicate a statistical significance.

### Survival analysis

Cutoff Finder (http://molpath.charite.de/cutoff) was used to determinate cutoff values for LIHC mRNA expression. Illuminahiseq_maseqv2-RSEM_genes_normalised RNA-seq data of NOX family genes were uploaded from a tab-separated files, and the rows represented patients and columns represented variables (http://molpath.charite.de/cutoff/load.jsp). The cutoff determination in Cutoff Finder was based on survival for significance based on the log-rank test for patient outcome (http://molpath.charite.de/cutoff/assign.jsp). The cumulative event (death) rate was calculated with the Kaplan-Meier method, using the time to the first event as the outcome variable. The criterion for the survival date for statistical analysis was the day from the date of operation to the date of death. Survival curves were compared by the log-rank test for high and low expression groups on each NOX gene family.

### Statistical analysis

Statistical analyses were performed using Prism software (v.5.0; GraphPad Prism Software, La Jolla, CA, USA) and the Statistical Package for Social Sciences for Windows (SPSS 13.0, Inc., Chicago, IL, USA). Distributions were compared between two groups by the t-test (or the Kolmogorov-Smirnov test when the expected frequency within any cell was <5) for continuous variables, and the χ^2^ test (or Fisher’s exact test when the expected frequency within any cell was <5 for categorical variables) for categorical variables. Distributions of the characteristics among three or more groups were compared by ANOVA. P < 0.05 was considered statistically significant.

## Electronic supplementary material

Supplementary Information
