# Supplementary material for: Gene expression of NOX family members and their clinical significance in hepatocellular carcinoma
Source: Sci Rep. 2017 Sep 11;7:11060. doi: 10.1038/s41598-017-11280-3 (PMC5593889; doi:10.1038/s41598-017-11280-3)
Supplement: Supplementary file 1 — Supplementary Information [file 41598_2017_11280_MOESM1_ESM.pdf]

Gene expression of NOX family members and their clinical significance in hepatocellular carcinoma

Hyuk Soo Eun[1,2†], Sang Yeon Cho[3,†], Jong Seok Joo[1,2], Sun Hyung Kang[1,2], Hee Seok Moon[1,2], Eaum Seok Lee[1,2], Seok Hyun Kim[1,2], Byung Seok Lee[1,2,*]

[1]*Division of Gastroenterology and Hepatology, Department of Internal Medicine, Chungnam National University Hospital, 282, Munwha-ro, Jung-gu, Daejeon, Republic of Korea,*

[2]*Department of Internal Medicine, School of Medicine, Chungnam National University, 266, Munwha-ro, Jung-gu, Daejeon, Republic of Korea,*

[3]*School of Medicine, Chungnam National University, 266, Munwha-ro, Jung-gu, Daejeon, Republic of Korea,*

[†]*These authors contributed equally to this work.*

[*]*Correspondence and requests for materials should be addressed to B.S. Lee (email:gie001@cnuh.co.kr)*

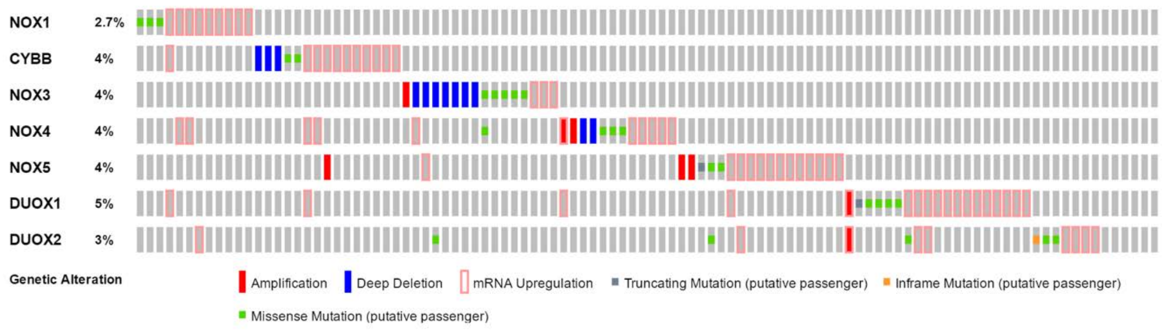

Supplementary figure 1. NOX family gene alterations in LIHC were obtained from the cBioportal database for cancer genomics.

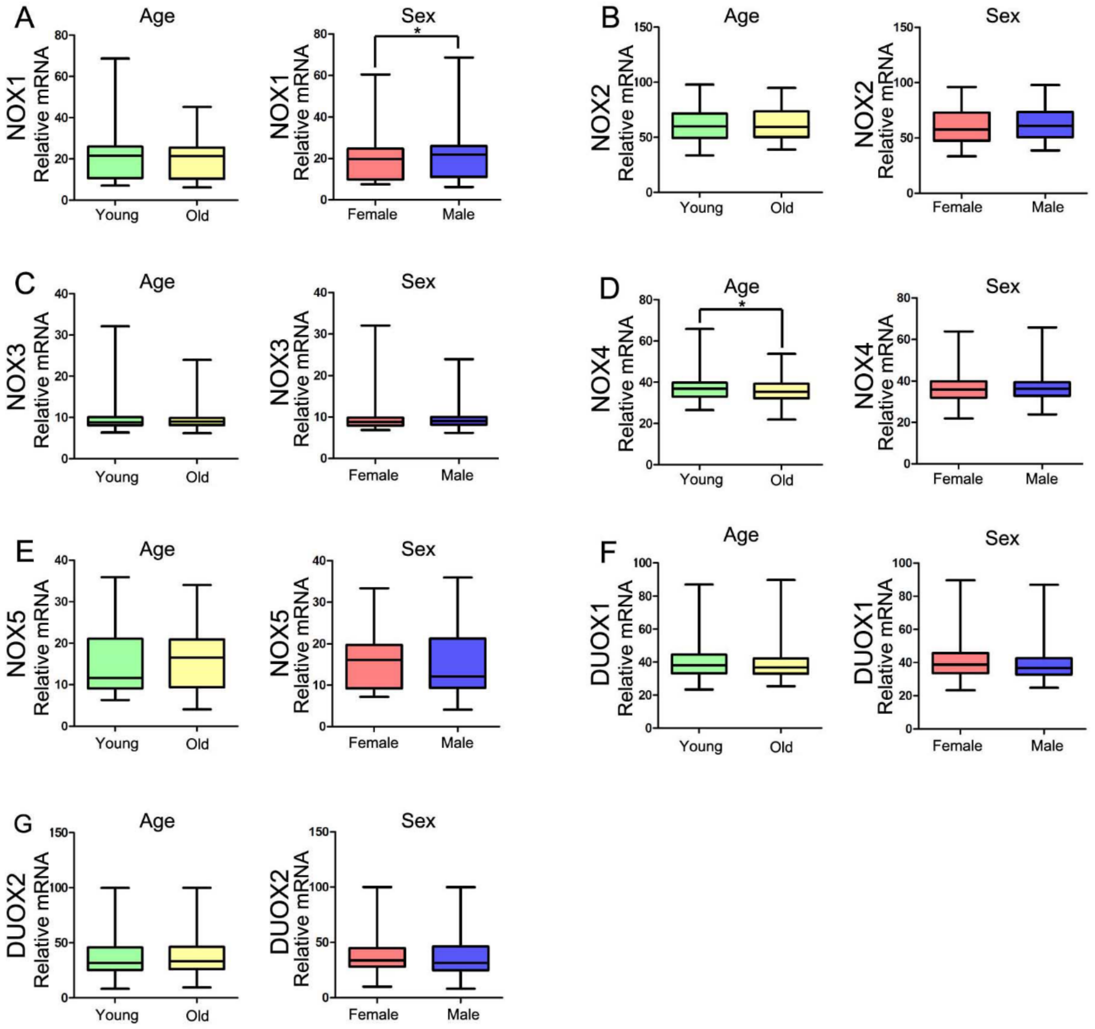

Supplementary figure 2. NOX family gene expression according to age and sex. Microarray data for NOX family gene expression in LIHC were obtained from the TCGA data portal. *$P < 0.05$ by one-way ANOVA.

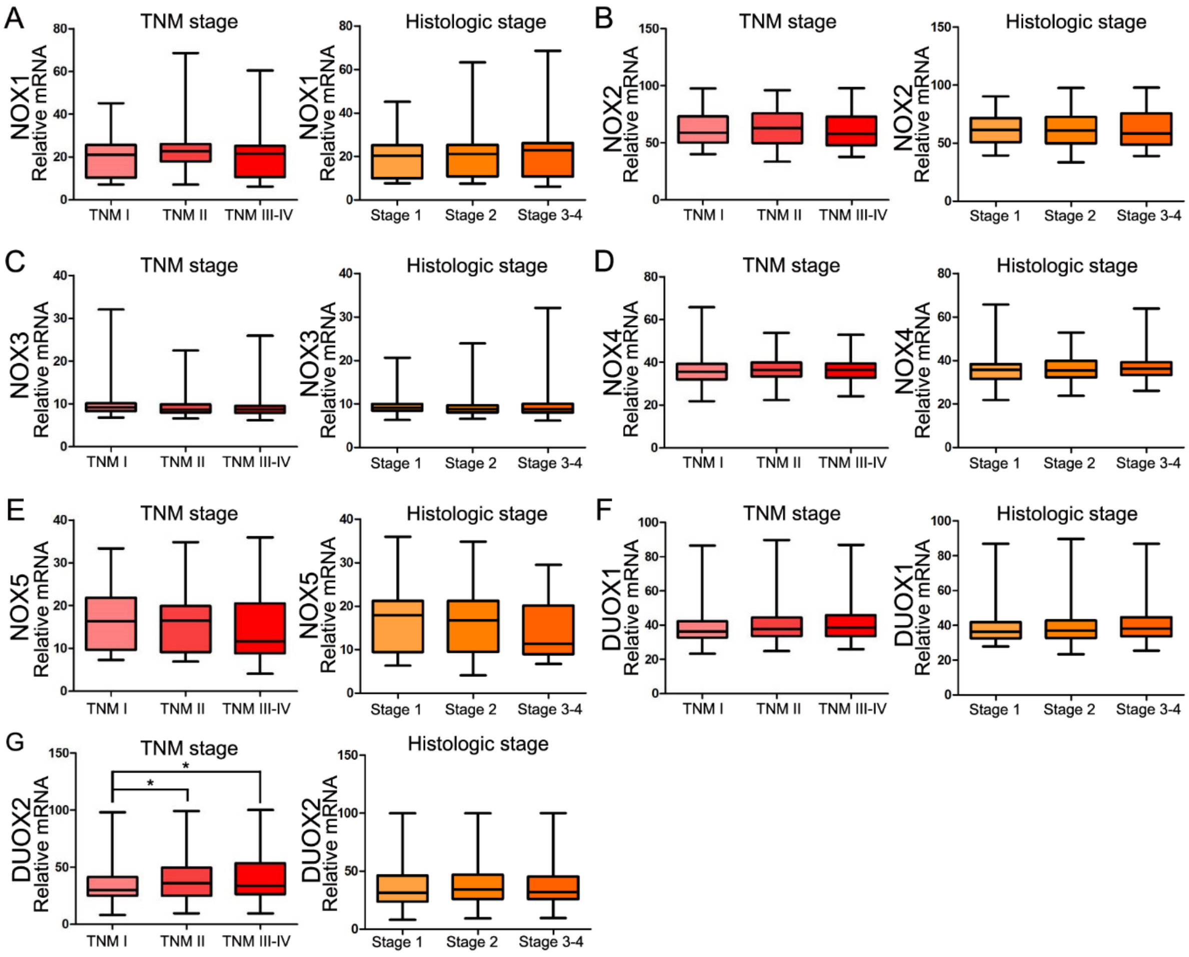

Supplementary figure 3. NOX family gene expression according to TNM stage and histologic grade. Microarray data for the NOX family gene expression in LIHC were obtained from the TCGA data portal. *P < 0.05 by one-way ANOVA.

Supplementary table 1. DAVID analysis results of GeneNeighbors of NOX family gene.

NOX1

Biological Process (BP)

Term	Count	%	P Value
GO:0006739~NADP metabolic process	2	2.2	0.03
GO:0006006~glucose metabolic process	3	3.3	0.04
GO:0051156~glucose 6-phosphate metabolic process	2	2.2	0.05

Cellular Component (CC)

Term	Count	%	P Value
GO:0005829~cytosol	35	38.5	0.00
GO:0005730~nucleolus	14	15.4	0.00
GO:0016020~membrane	21	23.1	0.00
GO:0005685~U1 snRNP	3	3.3	0.00
GO:0034715~pICln-Sm protein complex	2	2.2	0.03
GO:0005654~nucleoplasm	21	23.1	0.04
GO:0005737~cytoplasm	34	37.4	0.05

Molecular function (MF)

Term	Count	%	P Value
GO:0005515~protein binding	59	64.8	0.00
GO:0050661~NADP binding	3	3.3	0.01
GO:1990446~U1 snRNP binding	2	2.2	0.03

NOX2

Biological Process (BP)

Term	Count	%	P Value
GO:0006954~inflammatory response	17	17.5	0.00
GO:0007229~integrin-mediated signaling pathway	9	9.3	0.00
GO:0006955~immune response	13	13.4	0.00
GO:0050900~leukocyte migration	8	8.2	0.00
GO:0030593~neutrophil chemotaxis	6	6.2	0.00
GO:0030168~platelet activation	7	7.2	0.00
GO:0042113~B cell activation	4	4.1	0.00
GO:0006935~chemotaxis	6	6.2	0.00
GO:0002281~macrophage activation involved in immune response	3	3.1	0.00
GO:0002430~complement receptor mediated signaling pathway	3	3.1	0.00
GO:0035556~intracellular signal transduction	9	9.3	0.00
GO:0045579~positive regulation of B cell differentiation	3	3.1	0.00
GO:0007155~cell adhesion	9	9.3	0.00
GO:0043547~positive regulation of GTPase activity	10	10.3	0.00
GO:0048010~vascular endothelial growth factor receptor signaling pathway	4	4.1	0.01
GO:0006911~phagocytosis, engulfment	3	3.1	0.02
GO:0038095~Fc-epsilon receptor signaling pathway	5	5.2	0.02
GO:0002774~Fc receptor mediated inhibitory signaling pathway	2	2.1	0.02
GO:0045588~positive regulation of gamma-delta T cell differentiation	2	2.1	0.02
GO:0001812~positive regulation of type I hypersensitivity	2	2.1	0.02
GO:0006958~complement activation, classical pathway	4	4.1	0.02
GO:0030335~positive regulation of cell migration	5	5.2	0.02
GO:0030890~positive regulation of B cell proliferation	3	3.1	0.02
GO:0045766~positive regulation of angiogenesis	4	4.1	0.02
GO:0032729~positive regulation of interferon-gamma production	3	3.1	0.03
GO:0042110~T cell activation	3	3.1	0.03
GO:0010543~regulation of platelet activation	2	2.1	0.03
GO:0002309~T cell proliferation involved in immune response	2	2.1	0.03
GO:0034138~toll-like receptor 3 signaling pathway	2	2.1	0.04
GO:0045576~mast cell activation	2	2.1	0.04
GO:0050852~T cell receptor signaling pathway	4	4.1	0.05
GO:0050764~regulation of phagocytosis	2	2.1	0.05

Cellular Component (CC)

Term	Count	%	P Value

GO:0005886~plasma membrane	46	47.4	0.00
GO:0005887~integral component of plasma membrane	25	25.8	0.00
GO:0009986~cell surface	12	12.4	0.00
GO:0009897~external side of plasma membrane	8	8.2	0.00
GO:0005884~actin filament	5	5.2	0.00
GO:0070062~extracellular exosome	26	26.8	0.00
GO:0042629~mast cell granule	3	3.1	0.01
GO:0005602~complement component C1 complex	2	2.1	0.01

Molecular function (MF)

Term	Count	%	P Value
GO:0004872~receptor activity	9	9.3	0.00
GO:0046934~phosphatidylinositol-4,5-bisphosphate 3-kinase activity	4	4.1	0.00
GO:0042288~MHC class I protein binding	3	3.1	0.00
GO:0043325~phosphatidylinositol-3,4-bisphosphate binding	3	3.1	0.01
GO:0003779~actin binding	6	6.2	0.02
GO:0005096~GTPase activator activity	6	6.2	0.02
GO:0005085~guanyl-nucleotide exchange factor activity	4	4.1	0.02
GO:0004715~non-membrane spanning protein tyrosine kinase activity	3	3.1	0.02
GO:0004713~protein tyrosine kinase activity	4	4.1	0.03
GO:0005102~receptor binding	6	6.2	0.04
GO:0005515~protein binding	56	57.7	0.04
GO:0050839~cell adhesion molecule binding	3	3.1	0.04

NOX3

Biological Process (BP)

Term	Count	%	P Value
GO:0007186~G-protein coupled receptor signaling pathway	19	22.1	0.00
GO:0042742~defense response to bacterium	6	7.0	0.00
GO:0045087~innate immune response	6	7.0	0.02
GO:0031640~killing of cells of other organism	2	2.3	0.05

Cellular Component (CC)

Term	Count	%	P Value
GO:0045095~keratin filament	4	4.7	0.01
GO:0005576~extracellular region	14	16.3	0.01

Molecular function (MF)

Term	Count	%	P Value
GO:0004930~G-protein coupled receptor activity	17	19.8	0.00
GO:0004888~transmembrane signaling receptor activity	5	5.8	0.01

NOX4

Biological Process (BP)

Term	Count	%	P Value
GO:0001525~angiogenesis	15	15.6	0.00
GO:0030198~extracellular matrix organization	8	8.3	0.00
GO:0043542~endothelial cell migration	4	4.2	0.00
GO:0007155~cell adhesion	10	10.4	0.00
GO:0001666~response to hypoxia	6	6.3	0.00
GO:0007220~Notch receptor processing	3	3.1	0.00
GO:0010596~negative regulation of endothelial cell migration	3	3.1	0.00
GO:0016525~negative regulation of angiogenesis	4	4.2	0.00
GO:0030334~regulation of cell migration	4	4.2	0.01
GO:0030335~positive regulation of cell migration	5	5.2	0.01
GO:0071560~cellular response to transforming growth factor beta stimulus	3	3.1	0.03
GO:0003158~endothelium development	2	2.1	0.03
GO:0038063~collagen-activated tyrosine kinase receptor signaling pathway	2	2.1	0.03
GO:0016264~gap junction assembly	2	2.1	0.04
GO:0001938~positive regulation of endothelial cell proliferation	3	3.1	0.05

Cellular Component (CC)

Term	Count	%	P Value
GO:0005925~focal adhesion	11	11.5	0.00
GO:0031012~extracellular matrix	9	9.4	0.00
GO:0016324~apical plasma membrane	8	8.3	0.00
GO:0005886~plasma membrane	34	35.4	0.00
GO:0005788~endoplasmic reticulum lumen	6	6.3	0.00
GO:0009986~cell surface	9	9.4	0.01
GO:0005604~basement membrane	4	4.2	0.01
GO:0016469~proton-transporting two-sector ATPase complex	2	2.1	0.02
GO:0005587~collagen type IV trimer	2	2.1	0.03
GO:0016021~integral component of membrane	35	36.5	0.03
GO:0033180~proton-transporting V-type ATPase, V1 domain	2	2.1	0.04

Molecular function (MF)

Term	Count	%	P Value
GO:0005178~integrin binding	5	5.2	0.00
GO:0050840~extracellular matrix binding	3	3.1	0.01

GO:0005102~receptor binding	6	6.3	0.03

NOX5

Biological Process (BP)

Term	Count	%	P Value
GO:0001525~angiogenesis	12	12.9	0.00
GO:0030198~extracellular matrix organization	8	8.6	0.00
GO:0048514~blood vessel morphogenesis	3	3.2	0.00
GO:0035025~positive regulation of Rho protein signal transduction	3	3.2	0.01
GO:0043547~positive regulation of GTPase activity	9	9.7	0.01
GO:0007155~cell adhesion	8	8.6	0.01
GO:0016477~cell migration	5	5.4	0.01
GO:0003158~endothelium development	2	2.2	0.03
GO:0038084~vascular endothelial growth factor signaling pathway	2	2.2	0.03
GO:0030574~collagen catabolic process	3	3.2	0.04
GO:0010863~positive regulation of phospholipase C activity	2	2.2	0.05
GO:0000165~MAPK cascade	5	5.4	0.05

Cellular Component (CC)

Term	Count	%	P Value
GO:0031012~extracellular matrix	11	11.8	0.00
GO:0005578~proteinaceous extracellular matrix	10	10.8	0.00
GO:0005887~integral component of plasma membrane	21	22.6	0.00
GO:0016324~apical plasma membrane	8	8.6	0.00
GO:0005604~basement membrane	5	5.4	0.00
GO:0005886~plasma membrane	34	36.6	0.00
GO:0005925~focal adhesion	7	7.5	0.01
GO:0005826~actomyosin contractile ring	2	2.2	0.02
GO:0043235~receptor complex	4	4.3	0.02
GO:0009986~cell surface	7	7.5	0.05

Molecular function (MF)

Term	Count	%	P Value
GO:0005201~extracellular matrix structural constituent	6	6.5	0.00
GO:0019838~growth factor binding	3	3.2	0.01
GO:0005178~integrin binding	4	4.3	0.01
GO:0005021~vascular endothelial growth factor-activated receptor activity	2	2.2	0.03

GO:0003779~actin binding	5	5.4	0.04

DUOX1

Biological Process (BP)

Term	Count	%	P Value
GO:0090630~activation of GTPase activity	4	4.8	0.01
GO:0000278~mitotic cell cycle	3	3.6	0.01
GO:0031338~regulation of vesicle fusion	3	3.6	0.02
GO:0006886~intracellular protein transport	5	6.0	0.02
GO:0050665~hydrogen peroxide biosynthetic process	2	2.4	0.04

Cellular Component (CC)

Term	Count	%	P Value
GO:0005622~intracellular	14	16.7	0.01
GO:0000940~condensed chromosome outer kinetochore	2	2.4	0.03
GO:0005813~centrosome	6	7.1	0.04

Molecular function (MF)

Term	Count	%	P Value
GO:0019894~kinesin binding	4	4.8	0.00
GO:0017137~Rab GTPase binding	4	4.8	0.02
GO:0016174~NAD(P)H oxidase activity	2	2.4	0.03
GO:0008199~ferric iron binding	2	2.4	0.05

DUOX2

Biological Process (BP)

Term	Count	%	P Value
GO:0030855~epithelial cell differentiation	4	4.4	0.00
GO:0042743~hydrogen peroxide metabolic process	2	2.2	0.02
GO:0006906~vesicle fusion	3	3.3	0.03
GO:0050727~regulation of inflammatory response	3	3.3	0.04
GO:1903896~positive regulation of IRE1-mediated unfolded protein response	2	2.2	0.04
GO:0008285~negative regulation of cell proliferation	6	6.6	0.04

Cellular Component (CC)

Term	Count	%	P Value
GO:0005615~extracellular space	17	18.7	0.00
GO:0016021~integral component of membrane	39	42.9	0.00
GO:0016020~membrane	21	23.1	0.00
GO:0005886~plasma membrane	31	34.1	0.00
GO:0009986~cell surface	8	8.8	0.01
GO:0043235~receptor complex	4	4.4	0.02
GO:0070062~extracellular exosome	21	23.1	0.03
GO:0005783~endoplasmic reticulum	9	9.9	0.04
GO:0005789~endoplasmic reticulum membrane	9	9.9	0.04

Molecular function (MF)

Term	Count	%	P Value
GO:0005544~calcium-dependent phospholipid binding	4	4.4	0.00
GO:0017171~serine hydrolase activity	2	2.2	0.02
GO:0000978~RNA polymerase II core promoter proximal region sequence-specific DNA binding	6	6.6	0.02
GO:0001077~transcriptional activator activity, RNA polymerase II core promoter proximal region sequence-specific binding	5	5.5	0.02
GO:0045236~CXCR chemokine receptor binding	2	2.2	0.04

Supplementary table 2. GSEA results of NOX family gene.

NOX1

GeneOntology (GO)

Term	Size	ES	NES	NOM p-val
GO_KERATIN_FILAMENT	84	0.66	2.34	0.00
GO_INTERMEDIATE_FILAMENT	184	0.60	2.31	0.00
GO_TYPE_I_INTERFERON_RECEPTOR_BINDING	17	0.82	2.17	0.00
GO_INTERMEDIATE_FILAMENT_CYTOSKELETON	227	0.53	2.11	0.00
GO_POSITIVE_REGULATION_OF_PEPTIDYL_SERINE_PHOSPHORYLATION_OF_STAT_PROTEIN	21	0.74	2.04	0.00
GO_REGULATION_OF_PEPTIDYL_SERINE_PHOSPHORYLATION_OF_STAT_PROTEIN	21	0.74	2.04	0.00
GO_ORGANELLAR_RIBOSOME	72	0.60	2.04	0.00
GO_RIBOSOMAL_SUBUNIT	159	0.52	1.99	0.00
GO_MITOCHONDRIAL_TRANSLATION	105	0.55	1.98	0.00
GO_TRANSLATIONAL_TERMINATION	92	0.54	1.95	0.00
GO_REGULATION_OF_TYPE_I_INTERFERON_MEDIATED_SIGNALING_PATHWAY	39	0.62	1.94	0.00
GO_LARGE_RIBOSOMAL_SUBUNIT	93	0.53	1.92	0.00
GO_TRANSLATIONAL_ELONGATION	110	0.52	1.91	0.00

KEGG Pathway

Term	Size	ES	NES	NOM p-val
KEGG_REGULATION_OF_AUTOPHAGY	34	0.65	1.95	0.00
KEGG_RIBOSOME	87	0.45	1.60	0.01
KEGG_OXIDATIVE_PHOSPHORYLATION	116	0.44	1.59	0.00

NOX2

GeneOntology (GO)

Term	Size	ES	NES	NOM p-val
GO_ACTIVATION_OF_IMMUNE_RESPONSE	388	0.74	3.23	0.00
GO_ADAPTIVE_IMMUNE_RESPONSE	252	0.77	3.23	0.00
GO_IMMUNE_RESPONSE_REGULATING_CELL_SURFACE_RECEPTOR_SIGNALING_PATHWAY	286	0.75	3.20	0.00
GO_LEUKOCYTE_ACTIVATION	409	0.72	3.17	0.00
GO_POSITIVE_REGULATION_OF_CELL_ACTIVATION	284	0.74	3.15	0.00
GO_REGULATION_OF_CELL_ACTIVATION	454	0.71	3.13	0.00
GO_LEUKOCYTE_MIGRATION	259	0.75	3.13	0.00
GO_ANTIGEN_RECEPTOR_MEDIATED_SIGNALING_PATHWAY	170	0.77	3.12	0.00
GO_LYMPHOCYTE_ACTIVATION	338	0.72	3.11	0.00
GO_LEUKOCYTE_CHEMOTAXIS	117	0.81	3.11	0.00
GO_INFLAMMATORY_RESPONSE	445	0.70	3.11	0.00
GO_POSITIVE_REGULATION_OF_CELL_CELL_ADHESION	235	0.73	3.10	0.00
GO_REGULATION_OF_LEUKOCYTE_PROLIFERATION	202	0.74	3.06	0.00
GO_REGULATION_OF_CELL_CELL_ADHESION	371	0.70	3.03	0.00

KEGG Pathway

Term	Size	ES	NES	NOM p-val
KEGG_CHEMOKINE_SIGNALING_PATHWAY	188	0.72	2.94	0.00
KEGG_CYTOKINE_CYTOKINE_RECEPTOR_INTERACTION	264	0.68	2.88	0.00
KEGG_CELL_ADHESION_MOLECULES_CAMS	131	0.71	2.79	0.00
KEGG_T_CELL_RECEPTOR_SIGNALING_PATHWAY	108	0.72	2.74	0.00
KEGG_FC_GAMMA_R_MEDIATED_PHAGOCYTOSIS	95	0.72	2.69	0.00
KEGG_NATURAL_KILLER_CELL_MEDIATED_CYTOTOXICITY	132	0.66	2.61	0.00
KEGG_B_CELL_RECEPTOR_SIGNALING_PATHWAY	75	0.71	2.49	0.00
KEGG_TOLL_LIKE_RECEPTOR_SIGNALING_PATHWAY	102	0.63	2.41	0.00

Oncogenic Signature

Term	Size	ES	NES	NOM p-val
EGFR_UP.V1_UP	190	0.66	2.71	0.00
RAF_UP.V1_UP	191	0.59	2.42	0.00
KRAS.DF.V1_UP	191	0.56	2.31	0.00

NOX3

GeneOntology (GO)

Term	Size	ES	NES	NOM p-val
GO_KERATIN_FILAMENT	84	0.67	2.33	0.00
GO_INTERMEDIATE_FILAMENT	184	0.61	2.29	0.00
GO_TYPE_I_INTERFERON_RECEPTOR_BINDING	17	0.84	2.15	0.00
GO_INTERMEDIATE_FILAMENT_CYTOSKELETON	227	0.55	2.08	0.00
GO_MITOCHONDRIAL_TRANSLATION	105	0.58	2.06	0.00
GO_NATURAL_KILLER_CELL_ACTIVATION_INVOLVED_IN_IMMUNE_RESPONSE	24	0.74	2.05	0.00
GO_ORGANELLAR_RIBOSOME	72	0.61	2.04	0.00
GO_TRANSLATIONAL_TERMINATION	92	0.58	2.04	0.00
GO_CORNIFIED_ENVELOPE	43	0.65	2.04	0.00
GO_TRNA_PROCESSING	110	0.55	1.98	0.00
GO_RIBOSOMAL_SUBUNIT	159	0.52	1.94	0.00
GO_MITOCHONDRIAL_RESPIRATORY_CHAIN_COMPLEX_ASSEMBLY	74	0.55	1.87	0.00
GO_TRANSLATIONAL_ELONGATION	110	0.53	1.86	0.00
GO_DNA_PACKAGING_COMPLEX	98	0.53	1.86	0.00

KEGG Pathway

Term	Size	ES	NES	NOM p-val
KEGG_RNA_POLYMERASE	29	0.53	1.53	0.02
KEGG_RIBOSOME	87	0.44	1.51	0.00
KEGG_BASE_EXCISION_REPAIR	33	0.50	1.47	0.04
KEGG_OXIDATIVE_PHOSPHORYLATION	116	0.41	1.47	0.01

NOX4

GeneOntology (GO)

Term	Size	ES	NES	NOM p-val
GO_SISTER_CHROMATID_SEGREGATION	170	0.65	2.64	0.00
GO_MITOTIC_SISTER_CHROMATID_SEGREGATION	87	0.70	2.59	0.00
GO_CONDENSED_CHROMOSOME_CENTROMERIC_REGION	94	0.68	2.56	0.00
GO_SISTER_CHROMATID_COHESION	108	0.66	2.52	0.00
GO_MITOTIC_SPINDLE_ORGANIZATION	68	0.70	2.49	0.00
GO_DNA_PACKAGING_COMPLEX	98	0.65	2.47	0.00
GO_KINETOCHORE	111	0.63	2.46	0.00
GO_NUCLEAR_CHROMOSOME_SEGREGATION	218	0.58	2.46	0.00
GO_DNA_PACKAGING	179	0.59	2.45	0.00
GO_CHROMOSOME_CENTROMERIC_REGION	164	0.60	2.43	0.00
GO_METAPHASE_PLATE_CONGRESSION	40	0.75	2.43	0.00
GO_SPINDLE_MIDZONE	27	0.81	2.42	0.00
GO_DNA_REPLICATION_DEPENDENT_NUCLEOSOME_ASSEMBLY	31	0.77	2.42	0.00
GO_DNA_REPLICATION_DEPENDENT_NUCLEOSOME_ORGANIZATION	31	0.77	2.37	0.00
GO_MITOTIC_NUCLEAR_DIVISION	347	0.54	2.37	0.00
GO_PROTEIN_DNA_COMPLEX	162	0.58	2.37	0.00
GO_CHROMOSOMAL_REGION	310	0.54	2.36	0.00
GO_CHROMATIN_ASSEMBLY_OR_DISASSEMBLY	162	0.58	2.35	0.00
GO_ANAPHASE_PROMOTING_COMPLEX_DEPENDENT_CATABOLIC_PROCESS	77	0.65	2.34	0.00

KEGG Pathway

Term	Size	ES	NES	NOM p-val
KEGG_CELL_CYCLE	118	0.56	2.21	0.00
KEGG_DNA_REPLICATION	36	0.63	1.95	0.00
KEGG_HOMOLOGOUS_RECOMBINATION	26	0.62	1.82	0.00
KEGG_RIBOSOME	87	0.48	1.81	0.00
KEGG_SPLICEOSOME	114	0.44	1.69	0.00
KEGG_MISMATCH_REPAIR	23	0.58	1.68	0.01
KEGG_P53_SIGNALING_PATHWAY	67	0.44	1.58	0.01
KEGG_OXIDATIVE_PHOSPHORYLATION	116	0.38	1.47	0.00
KEGG_ECM_RECEPTOR_INTERACTION	83	0.37	1.37	0.04

NOX5

GeneOntology (GO)

Term	Size	ES	NES	NOM p-val
GO_REGULATION_OF_TYPE_I_INTERFERON_MEDIATED_SIGNALING_PATHWAY	39	0.73	2.28	0.00
GO_TYPE_I_INTERFERON_RECEPTOR_BINDING	17	0.88	2.23	0.00
GO_KERATIN_FILAMENT	84	0.61	2.15	0.00
GO_POSITIVE_REGULATION_OF_PEPTIDYL_SERINE_PHOSPHORYLATION_OF_STAT_PROTEIN	21	0.78	2.15	0.00
GO_REGULATION_OF_PEPTIDYL_SERINE_PHOSPHORYLATION_OF_STAT_PROTEIN	21	0.78	2.13	0.00
GO_DRUG_METABOLIC_PROCESS	39	0.67	2.12	0.00
GO_EPOXYGENASE_P450_PATHWAY	18	0.79	2.09	0.00
GO_INTERMEDIATE_FILAMENT	184	0.53	2.07	0.00
GO_MICROBODY_PART	92	0.56	2.05	0.00
GO_ACYLGLYCEROL_HOMEOSTASIS	29	0.69	2.05	0.00
GO_ALPHA_AMINO_ACID_CATABOLIC_PROCESS	94	0.58	2.04	0.00
GO_ORGANIC_ACID_CATABOLIC_PROCESS	202	0.51	2.04	0.00
GO_TRIGLYCERIDE_HOMEOSTASIS	29	0.69	2.02	0.00
GO_CARBOXYLIC_ACID_CATABOLIC_PROCESS	202	0.51	2.02	0.00

KEGG Pathway

Term	Size	ES	NES	NOM p-val
KEGG_REGULATION_OF_AUTOPHAGY	34	0.76	2.32	0.00
KEGG_DRUG_METABOLISM_CYTOCHROME_P450	71	0.63	2.21	0.00
KEGG_METABOLISM_OF_XENOBIOTICS_BY_CYTOCHROME_P450	69	0.61	2.07	0.00
KEGG_PEROXISOME	77	0.58	2.04	0.00
KEGG_FATTY_ACID_METABOLISM	40	0.63	2.01	0.00
KEGG_ASCORBATE_AND_ALDARATE_METABOLISM	24	0.69	1.96	0.00
KEGG_DRUG_METABOLISM_OTHER_ENZYMES	49	0.59	1.91	0.00
KEGG_GLYCINE_SERINE_AND_THREONINE_METABOLISM	31	0.63	1.90	0.00
KEGG_RIG_I_LIKE_RECEPTOR_SIGNALING_PATHWAY	71	0.54	1.86	0.00
KEGG_TRYPTOPHAN_METABOLISM	40	0.49	1.52	0.02

Oncogenic Signature

Term	Size	ES	NES	NOM p-val
PKCA_DN.V1_UP	167	0.40	1.54	0.00

DUOX1

GeneOntology (GO)

Term	Size	ES	NES	NOM p-val
GO_SISTER_CHROMATID_SEGREGATION	170	0.66	2.93	0.00
GO_MITOTIC_SISTER_CHROMATID_SEGREGATION	87	0.70	2.85	0.00
GO_CHROMOSOME_SEGREGATION	259	0.61	2.83	0.00
GO_CONDENSED_CHROMOSOME_CENTROMERIC_REGION	94	0.68	2.83	0.00
GO_NUCLEAR_CHROMOSOME_SEGREGATION	218	0.61	2.81	0.00
GO_MITOTIC_NUCLEAR_DIVISION	347	0.57	2.78	0.00
GO_CHROMOSOMAL_REGION	310	0.58	2.77	0.00
GO_CONDENSED_CHROMOSOME	183	0.61	2.77	0.00
GO_SISTER_CHROMATID_COHESION	108	0.66	2.76	0.00
GO_CHROMOSOME_CENTROMERIC_REGION	164	0.63	2.76	0.00
GO_KINETOCHORE	111	0.65	2.73	0.00
GO_MITOTIC_SPINDLE_ORGANIZATION	68	0.70	2.71	0.00
GO_ORGANELLE_FISSION	474	0.53	2.66	0.00
GO_DNA_PACKAGING	179	0.59	2.64	0.00
GO_CELL_DIVISION	434	0.53	2.63	0.00
GO_SPINDLE_MICROTUBULE	58	0.70	2.63	0.00
GO_DNA_REPLICATION	195	0.58	2.61	0.00
GO_DNA_CONFORMATION_CHANGE	257	0.56	2.61	0.00
GO_PROTEIN_DNA_COMPLEX	162	0.59	2.59	0.00
GO_CENTROMERE_COMPLEX_ASSEMBLY	45	0.72	2.57	0.00

KEGG Pathway

Term	Size	ES	NES	NOM p-val
KEGG_CELL_CYCLE	118	0.61	2.59	0.00
KEGG_DNA_REPLICATION	36	0.72	2.44	0.00
KEGG_HOMOLOGOUS_RECOMBINATION	26	0.68	2.15	0.00
KEGG_MISMATCH_REPAIR	23	0.62	1.87	0.00
KEGG_SPLICEOSOME	114	0.43	1.81	0.00
KEGG_P53_SIGNALING_PATHWAY	67	0.41	1.58	0.01
KEGG_BASE_EXCISION_REPAIR	33	0.46	1.51	0.02

DUOX2

GeneOntology (GO)

Term	Size	ES	NES	NOM p-val
GO_EXTRACELLULAR_STRUCTURE_ORGANIZATION	299	0.58	2.89	0.00
GO_EXTRACELLULAR_MATRIX_COMPONENT	123	0.56	2.57	0.00
GO_EXTRACELLULAR_MATRIX_DISASSEMBLY	74	0.61	2.49	0.00
GO_EXTRACELLULAR_MATRIX_STRUCTURAL_CONSTITUENT	74	0.61	2.47	0.00
GO_REGULATION_OF_LEUKOCYTE_MIGRATION	148	0.54	2.45	0.00
GO_MULTICELLULAR_ORGANISM_METABOLIC_PROCESS	91	0.57	2.43	0.00
GO_RHO_GUANYL_NUCLEOTIDE_EXCHANGE_FACTOR_ACTIVITY	74	0.59	2.38	0.00
GO_REGULATION_OF_RHO_PROTEIN_SIGNAL_TRANSDUCTION	105	0.55	2.38	0.00
GO_INTEGRIN_BINDING	105	0.55	2.37	0.00
GO_ANCHORING_JUNCTION	484	0.45	2.36	0.00
GO_CELL_CHEMOTAXIS	162	0.52	2.36	0.00
GO_EXTRACELLULAR_MATRIX	417	0.46	2.36	0.00
GO_MITOTIC_SPINDLE	54	0.62	2.35	0.00
GO_POSITIVE_REGULATION_OF_LEUKOCYTE_MIGRATION	109	0.54	2.34	0.00
GO_CELL_ADHESION_MOLECULE_BINDING	186	0.51	2.34	0.00
GO_REGULATION_OF_CHEMOTAXIS	179	0.50	2.34	0.00

KEGG Pathway

Term	Size	ES	NES	NOM p-val
KEGG_ECM_RECEPTOR_INTERACTION	83	0.58	2.36	0.00
KEGG_NOD_LIKE_RECEPTOR_SIGNALING_PATHWAY	62	0.57	2.22	0.00
KEGG_FC_GAMMA_R_MEDIATED_PHAGOCYTOSIS	95	0.51	2.16	0.00
KEGG_CELL_CYCLE	118	0.47	2.08	0.00
KEGG_FOCAL_ADHESION	198	0.43	2.03	0.00
